# Drug Cost-Effectiveness Assessments Require Standards for Rigor and Inclusion

**DOI:** 10.36469/001c.68194

**Published:** 2023-02-23

**Authors:** Tia Goss Sawhney, Neil Thakur

**Affiliations:** 1 Teus Health, LLC; 2 The ALS Association, Washington, DC

**Keywords:** drug prices, cost effectiveness, QALYs, data quality, Institute for Clinical and Economic Review (ICER)

## Abstract

The Institute for Clinical and Economic Review (ICER), a nonprofit, nongovernmental organization, is the predominant independent price assessor in the United States. ICER’s cost effectiveness assessments are increasingly being used to support health insurance coverage and healthcare policy decisions. ICER often does not apply rigorous data quality and inclusion criteria to either the assumptions embedded within their cost-effectiveness models or the data inputted into the models. Poor quality assumptions and data can lead to poor quality assessments.

ICER should re-evaluate their reliance on quality adjusted life-years and equal value of life years gained as measures of drug effectiveness, establish data quality and inclusiveness minimum standards, produce cost-effectiveness assessments only when the minimum data is available, and prominently report data quality and inclusion limitations. These changes will increase the rigor and inclusiveness of drug price assessments and support sustainable access to high-value care for all Americans.

How can we rationally and fairly determine drug prices in our complex American health system? Economists favor the cost-effectiveness assessment method, which compares a drug’s net cost (the price of the drug less any cost offsets, such as reduced hospitalizations) to measures of the drug’s effectiveness.

In many countries, drug price assessments are conducted by quasi-governmental organizations, such as the National Institute for Health and Care Excellence in the United Kingdom or Canada’s Drug and Health Technology Agency. In the United States, the Institute for Clinical and Economic Review (ICER), a nonprofit, nongovernmental organization, has increasingly become the predominant independent price assessor, offering “rigorous, transparent evidence reports” from the “US health care system perspective.”^1(p1,24)^

An ICER “value assessment” has two substantial sub-assessments: a comparativeness clinical effectiveness assessment and a long-term cost-effectiveness assessment. These sub-assessments are equally important, and weaknesses in either one will lead to an inaccurate value assessment that can distort prices or inappropriately limit access to new treatments.

The comparative clinical effectiveness assessment involves review of the methods, results, and generalizability of the drug’s randomized control trials and other studies to assess whether adding the drug to the standard of care will provide clinical benefit. These reviews apply predefined, rigorous assessment criteria and assign a letter grade to the quality of the evidence.^1^ While we and others sometimes disagree with specific conclusions drawn from this approach, we feel that the clinical effectiveness assessment methodology is generally sound.

In contrast, cost-effectiveness assessments often do not apply rigorous criteria to either the assumptions embedded within the model or the data inputted into the model. Poor quality assumptions and data can lead to poor quality assessments.

## Cost-Effectiveness Assessment Shortcomings

Some economists utilize quality-adjusted life-years (QALYs) as the primary measure for drug cost-effectiveness assessments. As has been extensively described by the National Council on Disability^2^ and others.^3,4^ QALYs have the unintentional effect of discriminating against people with disabilities, thereby limiting the generalizability of cost-effectiveness assessments that use these approaches.

ICER developed equal value of life-years gained (evLYGs) to counter the discriminatory bias of QALYs. However, evLYGs still draw from the same quality-of-life (QOL) utility measures that drive QALYs, particularly the EuroQol 5-dimension (EQ-5D) utility measure,^5^ and therefore are still inadequate for measuring the effectiveness of healthcare interventions for people with disabilities.

For example, because EQ-5D asks about a person’s ability to perform their “usual activities” and only evaluates a single day of health, a person whose usual life is highly restricted by their disease could have a maximum or near-maximum EQ-5D utility score. This measurement artifact would mean they have little to no QALYs to gain from treatment, skewing the assessment. Furthermore, perceptions of health and supports from long-term care and family differ widely across the world, making QOL a culturally specific construct. To assess the US value of a drug, an assessor first needs to engage US patients on how to best measure disease impact and drug effectiveness within the US health system. The answer may be several effectiveness measures rather than one or two.

A rigorous cost assessment for the US health system also requires data inputs that are inclusive and generalizable to all Americans who might take the drug under review. Good cost and QOL utility data are especially difficult to find for low-prevalence diseases. In the absence of high-quality data, ICER has relied on data that are old, non-US, extracted from small and atypical populations, and generally not reflective of US healthcare and populations for both the cost and effectiveness portions of their cost-effectiveness assessments.

For example, a 2022 assessment reviewed two newly approved drugs for the treatment of amyotrophic lateral sclerosis (ALS).^6^ ALS is a rare, disabling, and deadly disease affecting about 30 000 Americans. The comparative clinical effectiveness assessment carefully reviewed and graded the quality and generalizability of the relevant clinical trials, including issues such as quality of measurement, sample size, and inclusion. For the cost-effectiveness assessment inputs, however, there was no such review.^6^

The ALS cost-effectiveness assessment model inputs included QOL data collected between 2009 and 2012 from a cohort of 214 ALS patients in the United Kingdom, many of whom did not respond to QOL questions as their disease progressed; US commercial health insurance data for services provided between 2008 and 2011, even though almost all Americans with ALS have been Medicare-eligible since 2020; and 2013 caregiving costs for 159 ALS patients in South Korea. Costs and burdens of illness from health systems like the United Kingdom and South Korea are not generalizable to the United States for a disease like ALS, which means the price recommendations for the US market in the report were based on cost-effectiveness data not generalizable to the United States.

We have seen similar lack of data standards in other cost-effectiveness assessments, including where foreign or dated source data were inappropriately generalized to contemporary Americans of color. For example, lupus nephritis (LN) is a rare disease with higher rates of prevalence and worse outcomes in Black and Hispanic Americans than in White, non-Hispanic Americans. A 2021 LN drug cost-effectiveness analysis, however, relied on results from international drug trials that uniformly underrepresented Black patients and mostly underrepresented Hispanic patients. It also used QOL utility data from 18 Thai patients with LN and 339 Swedish patients with systemic lupus erythematosus (a related disease), and estimated end-stage renal disease and mortality events for 86 US patients tracked from 1981 to 1988 (before the advent of biologics).^7^ The price assessment relied on data that clearly cannot be generalized to a contemporary US LN population.

## Recommendations

The lack of standards for cost-effectiveness assessments and use of nongeneralizable data can distort the benefits of new treatments and undermine the progress the scientific community has been making to increase the inclusivity and rigor of clinical trials. Lack of rigor and inclusivity in cost-effectiveness assessments can also lead to ineffective health insurance coverage and poor-quality healthcare policy decisions.

Fortunately, ICER makes a conscientious effort to learn and improve over time and routinely reviews their value assessment framework. When ICER updates their value assessment framework in 2023, we recommend they:

1. Convene US stakeholders to reevaluate its reliance on QALYs and evLYG as measures of drug effectiveness.
2. Extend the value assessment framework to require a rigorous, pre-registered process for assessing the data quality and inclusion of cost-effectiveness model inputs.
3. Develop a minimum evidence standard for cost-effectiveness assessment.
4. Prominently report the inevitable data quality and inclusion limitations of their assessments, even for data that are of overall sufficient quality.
5. Recognize that it may not be possible to meet the minimum evidence standard for every disease without additional data collection.

When data available for an assessment are of insufficient quality, an assessment should be considered incomplete, and the report should describe the data required to complete the assessment. These changes will increase rigor and inclusiveness of drug price assessments and support our shared goals of sustainable access to high-value care for all Americans.

### Disclosures

N.T. is employed by The ALS Association, and ALS drugs were recently assessed by ICER. T.G.S. has assisted several clients, including The ALS Association, with their comments on ICER assessments.
